# Advancing therapeutic approaches for agitation: A neurorelational perspective

**DOI:** 10.1002/trc2.70333

**Published:** 2026-10-14

**Authors:** Kathy Y. Liu, George Salaminios, Mara Mather, Robert Howard

**Affiliations:** ^1^ Division of Psychiatry University College London London UK; ^2^ Research Department of Clinical, Educational and Health Psychology University College London London UK; ^3^ Research Department British Association of Counselling and Psychotherapy Lutterworth UK; ^4^ Leonard Davis School of Gerontology University of Southern California Los Angeles California USA; ^5^ Department of Psychology University of Southern California Los Angeles California USA; ^6^ Department of Biomedical Engineering University of Southern California Los Angeles California USA

**Keywords:** agitation, caregiver, dementia, mentalization, noradrenergic

## Abstract

Agitation is a common, distressing, and difficult‐to‐treat symptom in dementia, linked to emotional dysregulation and interpersonal stress. Degenerative and compensatory changes in the locus coeruleus–noradrenergic (LC–NA) system have been implicated in agitation in Alzheimer's disease. However, substantial interindividual variability in the emergence of agitation over the course of dementia suggests that neuropathological changes alone are unlikely to fully account for individual differences in susceptibility to agitation. We explore how mentalizing capacity (the ability to understand one's own and others’ thoughts, feelings, beliefs, and intentions) in both care recipients and caregivers may influence agitation propensity. Mentalizing supports adaptive emotion regulation and may underpin person‐centered care, but becomes less effective under conditions of heightened arousal and interpersonal stress. It is proposed that LC–NA regulation of arousal may influence the capacity to sustain mentalizing under stress, potentially contributing to agitation and increasing the likelihood of escalation of care. Notably, mentalizing may be compromised by different processes in care recipients and caregivers. In care recipients, neurodegeneration may impair mentalizing, potentially in part through dysregulation of the LC–NA system. In caregivers, distress, burden, and emotionally challenging interactions may undermine the capacity to maintain mentalizing. As disease progression is likely to increasingly constrain mentalizing capacity in care recipients, we consider pharmacological modulation of care recipients’ LC–NA function and psychosocial interventions to strengthen caregiver mentalizing as complementary and potentially modifiable targets for intervention. This conceptualization is best understood within a relationship‐centered care perspective, which extends beyond person‐centered care by emphasizing care as embedded in the dynamic relationships between caregivers and care recipients, rather than focusing solely on individual patient preferences. Taken together, these considerations inform a neurorelational framework for understanding and addressing agitation, integrating neurobiological and socio‐emotional perspectives to provide a basis for personalized intervention approaches to potentially mitigate the effects of neurodegeneration on “neuropsychiatric reserve.”

## Highlights

- Agitation in dementia is related to emotion dysregulation.
- Neurodegenerative and mentalizing processes influence agitation propensity.
- Neurobiological and socio‐emotional perspectives could be integrated.
- Therapeutic approaches should consider both care recipients and caregivers.

## 1 INTRODUCTION

Agitation in the context of cognitive impairment is defined as a syndrome underpinned by emotional distress, manifesting as a range of behaviors classed as physical or verbal aggression and/or motor overactivity (e.g., restlessness, pacing, repetitive vocalizations).^1^ In long‐term care, these behaviors commonly occur during intimate care activities, such as dressing or toileting.^2^ Prevalence estimates vary between 5% to 88%, likely related to different definitions, populations, and settings, with a median of ≈ 30%.^3^ Among neuropsychiatric symptoms in Alzheimer's disease (AD), agitation causes the most caregiver burden,^4^ reduces quality of life,^5^ and significantly increases care costs and institutionalization risks.^6^ It is also associated with faster cognitive and functional decline and increased dementia risk in individuals with mild cognitive impairment (MCI) and preclinical AD.^7^ Pharmacological treatments are limited but necessary for individuals with severe symptoms unresponsive to non‐pharmacological approaches. Atypical antipsychotics offer modest efficacy (Cohen *d* = –0.21, 95% confidence interval [CI] –0.30 to –0.12),^8^ but carry serious risks, including sedation, parkinsonism, and increased mortality. With rising global prevalence of dementia, including a concomitant increase in caregivers, there is a pressing need for safe, targeted treatments for agitation.

Agitation is widely considered to communicate lack of understanding or unmet needs in the context of neurodegenerative decline, in which the individual may struggle to explain or understand their unmet physical or emotional needs.^2^ Contributing factors span biological, psychological, and/or social/environmental domains, such as pain, delirium, sensory impairment, and mood and psychotic disorders. More severe neuropathology, particularly AD severity,^9^ is associated with higher agitation prevalence,^3^ but agitation is not just a product of worsening cognitive status, as early changes in personality and behavior occur in one third of cognitively normal older people and approximately half of people with MCI.^10^ Agitation and other related neuropsychiatric symptoms such as impulsivity are detectable at higher levels in MCI and cognitively unimpaired individuals with a greater risk of developing dementia.^7^ Longitudinal studies show that while agitation severity increases with time, its prevalence remains relatively stable^3^, ^11^ and a proportion of individuals do not develop agitation despite increasing dementia severity.^11^ These findings suggest that disease severity alone is unlikely to fully account for individual differences in agitation susceptibility. In this article, we propose that individual differences in emotion regulation capacity, in both care recipients and caregivers, may contribute to variation in agitation propensity in dementia. We consider how premorbid emotion regulation may interact with neurodegenerative change in care recipients and with interpersonal stress and burden experienced by caregivers. We further propose that integrating these perspectives within a neurorelational framework may help explain how these processes interact within the caregiving relationship and identify potentially modifiable targets for intervention.

## 2 THE RELATIONSHIP BETWEEN EMOTION REGULATION AND AGITATION PROPENSITY

Emotion regulation has been broadly conceptualized as the processes through which individuals influence which emotions they have, when they have them, and how they experience and express them.^12^ We define emotion regulation here more specifically as the neurally controlled change in one's cognitive/bodily state from a more intensely emotional to a less intensely emotional state.^13^, ^14^ This process is a critical component of healthy psychological and social functioning and is considered a developmental process involving brain regions that integrate cognitive, behavioral, and physiological (autonomic) components.^15^ Explicit and implicit emotion processing involves a hierarchical relationship between these regions, in which bottom‐up signals of visceral changes from “lower level” (e.g., subcortical) structures interact with top‐down signals from “higher level” (e.g., prefrontal) structures, with higher levels representing more sophisticated cognitive and conscious processes, for example, meta‐cognition, that are more strongly associated with emotion regulation versus generation in certain contexts.^16^ For example, both the amygdala and prefrontal cortex (PFC) contribute to emotion regulation, with the former initiating faster and more automatic appraisals of bodily state (in response to present metabolic needs) and the latter initiating slower cognitive appraisal mechanisms (in response to present and future metabolic needs).^15^ Individual differences in emotion regulation capacity may be related to the nature and degree of neurovisceral integration, particularly during sensitive periods of human development, as stress and adversity during infancy or adolescence may influence or interfere with these processes.^17^ These individual differences in emotion regulation capacity may then be further impacted by neurodegenerative processes that lead to dementia.

### 2.1 Impact of AD‐related neuropathology on emotion regulation

Agitation propensity in AD is linked to structural and functional differences in key emotion‐regulation regions, including the PFC, anterior cingulate cortex (ACC), insula, amygdala, and hippocampus,^18^ which are components of broader networks such as the default mode network.^19^ Network‐level dysfunction in these circuits may disrupt coordinated emotion regulation and increase agitation vulnerability.^20^ Neurodegenerative processes, particularly early abnormal tau accumulation in the noradrenergic (NA) locus coeruleus (LC), which is associated with compensatory overactivity in the central NA system, are associated with agitation propensity from preclinical stages when most individuals are cognitively normal^21^, ^22^, ^23^, ^24^, ^25^ (described further in section 3.2). Agitation across the AD spectrum is associated with tau pathology, with greater burden linked to increased agitation, alongside broader neurobiological contributions (see Section S1 in supporting information).

### 2.2 Premorbid emotion regulation capacity and neuropsychiatric reserve

Differences in agitation propensity between individuals with dementia may stem from differences in premorbid emotion regulation capacity, akin to the concept of cognitive reserve. A life course and developmental perspective is therefore relevant to identify potentially modifiable risk factors that could influence “neuropsychiatric reserve.” While evidence is limited, studies indicate that prior psychological trauma^26^ and pre‐morbid neuroticism^27^ increase agitation risk in dementia. However, it can be challenging to interpret the causal direction between midlife psychological measures and dementia‐related agitation because AD neuropathology can accumulate decades before clinical symptoms, and AD dementia has been associated with increased neuroticism.

While further studies are needed to develop and test the “neuropsychiatric reserve” hypothesis, extensive evidence exists on factors influencing emotion regulation capacity. For example, the intensity of stimuli and the individual's social and cultural context, cognitive capacity, and developmental trajectory can influence the selection of emotion regulation strategies and their effectiveness.^28^ An enduring factor underlying emotion regulation capacity is an individual's context‐dependent attachment behavior system or strategy,^29^ which largely develops as an adaptation to their immediate social environment in infancy and childhood.

Attachment strategies are context‐specific behaviors that attempt to restore real or symbolic closeness to external or internalized attachment figures and a sense of security, in response to perceived threat of loss or security. They are typically conceptualized as comprising secure and insecure patterns, with insecure attachment including avoidant and ambivalent orientations.^30^ Attachment patterns remain relatively consistent throughout life and are relevant to caregiving and care‐receiving in older adults, who commonly experience fear of loss, separation, and vulnerability in relation to aging and chronic or debilitating illness. Attachment security can be measured and classified into attachment types using self‐ or informant‐reported interviews or questionnaires. Adult attachment can also be conceptualized dimensionally along two orthogonal axes, attachment anxiety and attachment avoidance, which correspond to hyperactivation and deactivation strategies, respectively.^31^ A more secure attachment pattern is associated with more effective use of emotion regulation strategies such as cognitive reappraisal. In contrast, activation of insecure attachment behavior systems is associated with maladaptive emotion regulation strategies that contribute vulnerability to psychopathology. Ambivalent individuals show heightened proximity seeking, distress, and vigilance, whereas avoidant individuals suppress or inhibit negative emotions.^31^

Only a small number of studies have examined attachment patterns and relationships in dementia. These found that attachment patterns could represent risk (or protective) factors for agitation symptoms.^32^, ^33^ Compared to individuals with dementia who had high levels of attachment security, those characterized by insecure attachment showed more behavioral and psychological symptoms.^33^ Caregivers’ attachment strategies, which are linked to reported levels of caregiver burden, commitment, and quality of caregiving,^33^ may also interact with care recipients’ attachment strategies to influence each other's responses to stress or perceived threat within the attachment relationship, predisposing to or triggering agitation symptoms.^32^, ^34^ Mitigating attachment insecurity in caregivers and/or care recipients may represent an as yet untargeted approach to reduce agitation propensity in older individuals experiencing cognitive and functional decline, which is associated with threat of loss and separation from attachment figures, activation of attachment needs, and emergence of “attachment seeking” behaviors.^35^

## 3 EMOTION REGULATION MARKERS AND TREATMENT APPROACHES IN AGITATION

Research into the psychological and neurobiological mechanisms linking attachment security and emotion regulation capacity has identified potential mediating factors, which may serve as risk or stratification markers for agitation propensity in dementia and inform novel treatment approaches.

### 3.1 Mentalizing capacity

Mentalizing represents an important cognitive–affective capacity relevant to emotion regulation and interpersonal functioning. It develops within secure attachment relationships through caregivers’ capacity to understand and respond to the child's feelings, needs, and intentions, fostering the child's own reflective functioning.^36^ Mentalizing enables behaviors to be understood in terms of underlying mental states and supports adaptive emotion regulation during interpersonal interactions. Under conditions of heightened stress or perceived threat to attachment relationships, however, individuals may shift from controlled mentalizing (supported predominantly by higher cortical processes) to more automatic or non‐mentalizing states (involving lower subcortical processes), which are less effective at downregulating arousal and reducing social avoidance^37^, ^38^ (Figure 1). This dependence of mentalizing on the ability to regulate arousal provides a potential point of convergence with LC–NA system function, which is considered further below.

**FIGURE 1:**
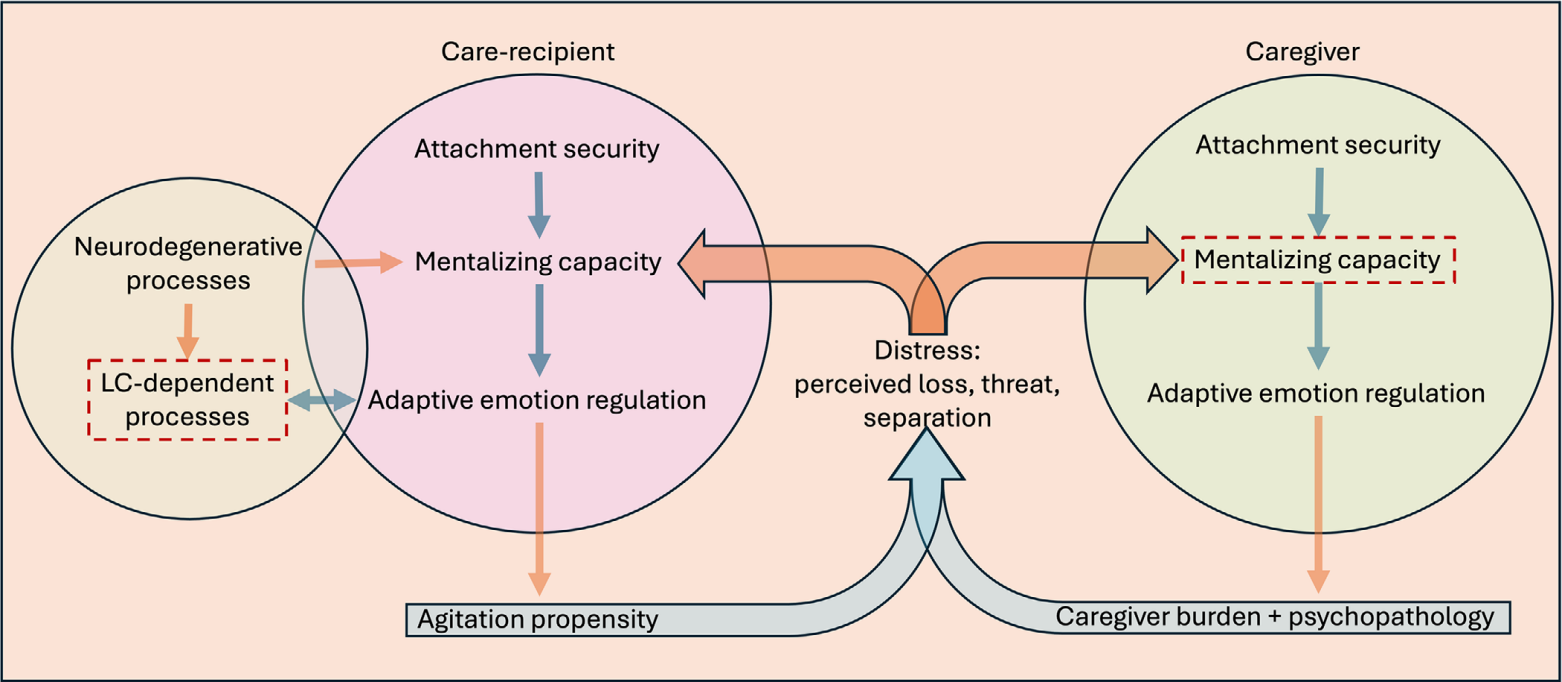
An integrated neurorelational model of agitation propensity in dementia. The abilities of both care recipients and caregivers to respond adaptively to acute interpersonal distress in the context of dementia are influenced by their mentalizing capacity, which is rooted in attachment security and is proposed to mediate its influence on emotion regulation. Neurodegenerative processes, including changes affecting the locus coeruleus (LC)‐noradrenaline system, may additionally impact these abilities in the care recipient, which may be indexed by physiological markers. The relational nature of this model reflects how emotional distress from care‐recipient agitation and caregiver burden or psychopathology can impair mentalizing in both parties, potentially creating a feedback loop that intensifies agitation and caregiver distress. Such escalating distress is also associated with higher care costs and an increased likelihood of institutionalization. LC‐dependent processes in the care recipient and mentalizing capacity in the caregiver (red dashed lines) represent potentially modifiable domains through noradrenergic‐lowering agents and mentalization‐enhancing approaches, respectively. Blue and orange arrows represent positive and negative associations between constructs, respectively.

Mentalizing capacity can be assessed using interview‐based or self‐report measures of reflective functioning and performance‐based theory of mind (ToM) tasks. Although ToM overlaps with mentalizing, it represents a narrower construct focused primarily on understanding others’ mental states, whereas mentalizing also encompasses affective and self‐reflective processes. Greater mentalizing is linked to more adaptive emotion regulation and stress resilience^39^ and better psychotherapeutic outcomes,^40^ while impaired mentalizing is implicated across a range of psychopathological conditions. Mentalization‐based interventions have shown benefits in psychiatric populations, including those at increased risk of psychopathology, and in caregiver–child relationships, supporting mentalizing as a modifiable therapeutic target.^41^, ^42^, ^43^

Although reflective functioning has not been directly investigated in individuals with dementia, ToM studies generally support that the capacity to sustain controlled mentalizing declines with disease progression. Poorer ToM performance has also been linked to greater neuropsychiatric symptom propensity.^44^ In addition, impaired ToM in care recipients has been associated with impaired ToM and greater perceived burden in caregivers, particularly in frontotemporal dementia,^45^ supporting that dementia‐related behavioral and emotional changes may also adversely affect caregivers’ mentalizing capacity.

As the individual with dementia's ability to understand and communicate their needs becomes increasingly impaired, caregiver mentalizing becomes increasingly important for interpreting and responding to the care recipient's internal mental states. Although mentalizing may influence agitation in care recipients, neurodegenerative processes may limit the extent to which their mentalizing capacity can be modified, making caregiver mentalizing a potentially more feasible target for intervention. In contrast to the secure attachment relationship between a parental caregiver and their infant or child, the emotional contact between an individual with dementia and their caregiver (most of whom are family members), is often contextualized by feelings of loss, anger, and for the caregiver, frustration and guilt, which can be difficult to tolerate^35^ and can impair mentalizing processes.^2^ In long‐term care settings, additional structural and procedural constraints, perceived lack of support and training, and a culture of fear and scrutiny can make maintaining reflective functioning more difficult.^2^ A failure, breakdown, or distortion of caregiver mentalizing, alongside associated difficulties in emotion regulation could underlie implicitly driven interactions that undermine person‐centered care. These include negative or “malignant” positioning of individuals with dementia by caregivers as someone who is seen (and then treated) as defective or worthy of punishment. This can lead to interpersonal conflict that will further validate the initial positioning as the person with dementia or caregiver may react negatively to it. These processes could contribute to the observation that caregivers may prefer or prioritize task‐ versus patient‐orientated activities (or physical vs. emotional needs), in part due to a disinclination to offer emotional contact, which can lead to individuals with dementia feeling unwanted or not understood, thus increasing agitation risk. Distressing neuropsychiatric symptoms such as agitation significantly increase levels of caregiver burden and distress and are likely to negatively impact caregivers’ capacity for controlled mentalizing. This may create a bidirectional cycle in which caregiver distress and emotionally challenging interactions further impair mentalizing and increase the likelihood of responses that escalate care recipient distress and agitation, potentially increasing the risk of institutionalization^46^ (see Figure 1). It is therefore relevant to consider caregiver mentalizing as a potentially modifiable component of the caregiving relationship. Supporting caregivers to reflect on their own and the care recipient's mental states, while regulating their own emotional responses, may sustain more adaptive interactions as dementia progresses. Indeed, better mentalizing abilities among long‐term care staff has been associated with more emotionally responsive behaviors toward and greater engagement from long‐term care residents with dementia.^47^ Conversely, higher levels of expressed emotion, through which caregivers express critical comments, show hostility, or engage in emotional overinvolvement, have been associated with dementia‐related neuropsychiatric symptoms including agitation.^48^ Studies of mentalization‐based interventions have shown improved caregiver outcomes,^49^ but it remains unknown if these can reduce agitation propensity as none have targeted or measured neuropsychiatric symptoms in individuals with dementia.

Supervised person‐centered interventions that focus on communication with people with dementia and help staff to understand and fulfil their wishes represent the most effective non‐pharmacological interventions for dementia‐related agitation.^50^ Although these interventions have not explicitly targeted caregivers’ mentalizing capacity, this might be a core component underlying their effectiveness through improved reflective function, emotion regulation, and interpersonal relationships; in other words, care that is “relationship centered.” While person‐centered care has been widely adopted, it is usually operationalized in terms of individual patient preferences and involvement. In contrast, a relationship‐centered care perspective emphasizes the dynamic relationships between caregivers and care recipients and aligns with the original, broader definition of person‐centered care, which situates care within the relational context between “carers” and “cared for.”^51^ In support of this, supervised person‐centered care interventions show greater effects than activities and music and sensory interventions and the individualization of activities has no clear additional benefit over prescribed activities.^50^

Cognitive–affective processes underlying mentalizing capacity and emotion regulation are also linked to neurobiological processes, particularly those related to the central NA system. This system thus provides a complementary neurobiological perspective on individual differences in emotion regulation and agitation vulnerability through its role in arousal, stress responsivity, and adaptive cognitive and behavioral responses to stress.

### 3.2 Central NA system (over)activity and autonomic reactivity

At a neurobiological level, several systems are proposed to contribute to the formation and expression of attachment patterns, particularly in response to perceived threat. Of these, the central NA system, mainly comprising cortical and subcortical projections to and from the pontine LC, is strongly implicated in cognitive and behavioral processes underlying adaptive responses to stress and salient stimuli.^52^ The LC is a major wakefulness‐promoting nucleus and promotes and maintains arousal by exerting an overall excitatory influence on the cortex. The LC also promotes overall sympathetic activation and parasympathetic inhibition via an autonomic network, inhibits pain sensation through a nociceptive modulation network, and generates or mediates fear/anxiety responses via strong reciprocal connections with the amygdala.^53^

The LC–NA system is in a key position to respond adaptively to changing environmental demands through two complementary modes of activity: phasic, higher‐frequency bursts elicited by salient external stimuli and internal decision‐making processes, which are superimposed on a lower‐frequency tonic discharge pattern positively correlated with arousal state and and modulated by input from frontal structures.^54^ NA receptor subtypes vary in their affinity for NA (a_2_> a_1_> β),^55^, ^56^ which allows for regional differences in how LC–NA release affects activity depending on local representation of receptor subtypes and local levels of glutamate activity which can stimulate local release of NA and therefore influence which receptor subtypes are targeted.^57^ For PFC‐mediated performance on tasks requiring focused attention, the effect of tonic LC–NA activity is proposed to follow an inverted U dose–response curve^56^ (Figure 2). Adaptive behavior is promoted by switching between the phasic mode, during which a burst of LC activity (accompanied by intermediate LC tonic activity) optimizes PFC‐mediated task‐appropriate and attentive behavior via higher affinity ɑ_2_‐receptors, and the tonic mode, during which high LC tonic activity (accompanied by decreased phasic LC activity) decreases PFC‐mediated attentional performance with task disengagement via lower affinity ɑ_1_‐receptors. Frontal structures may modulate LC tonic activity to facilitate or disengage from task‐specific processes to optimize utility (i.e., benefits vs. costs associated with performance).^54^

**FIGURE 2:**
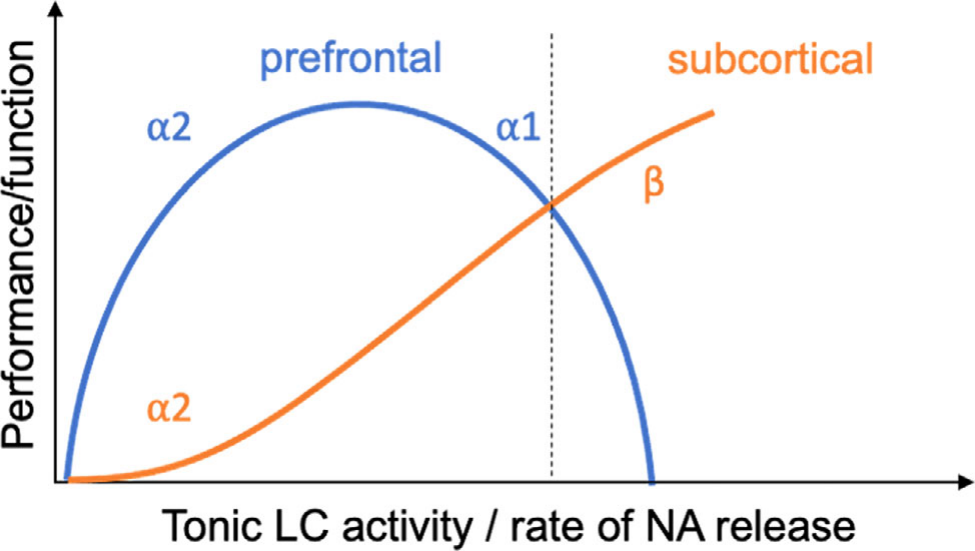
Differential effects of noradrenaline on prefrontal and subcortical function. The figure represents the effects of LC–NA activity on prefrontal (blue line) and subcortical (e.g., hippocampus and amygdala) (orange line) function. Intermediate NA release leads to optimal PFC‐mediated performance on tasks requiring focused attention via activation of higher affinity ɑ_2_‐receptors. In contrast, activation of ɑ_2_‐receptors in subcortical regions has an inhibitory effect. High NA release decreases PFC performance and corresponds to distractible (stress) states via engagement of lower affinity ɑ_1_‐receptors. In contrast, high NA release optimizes subcortical function via lower affinity β‐receptors. The dotted line represents the level of LC–NA activity at which a cognitive “switch” occurs between prefrontal and subcortical functions. LC, locus coeruleus; NA, noradrenergic; PFC, prefrontal cortex.

While PFC connectivity and performance is optimized by moderate NA levels (via ɑ_2_‐receptors) and compromised by high NA levels (via ɑ_1_‐receptors), posterior brain regions such as the amygdala and hippocampus show the opposite effect, that is, strong tonic NA release from LC optimizes hippocampal/amygdala performance via lower affinity β‐receptors, and ɑ_2_‐receptor activation has an inhibitory effect^58^ (Figure 2). Thus, in addition to modulating local (i.e., phasic vs. tonic) modes of neural activity, LC–NA system activity can shift the regional balance between neural processes in cortical (e.g., PFC) and subcortical (e.g., amygdala, hippocampus) structures, in response to environmental/internal demands.

The LC–NA system plays a key role in promoting behavioral adaptation to unexpected situations by promoting cognitive (attentional) shifts through neural networks “resets.”^54^, ^59^ The LC–NA system‐mediated adaptive switch between modes of behaviors subserved by either mainly PFC or posterior and subcortical regions parallels the proposed switch between controlled and non‐mentalizing states in response to stress or threat to attachment relationships. A lower threshold for this switch, linked to more insecure attachment behavior systems,^29^ may plausibly arise through elevated tonic LC activity and/or increased sensitivity of target adrenoceptors. LC activity is also influenced by stress‐responsive neuromodulatory systems, and its role in prioritizing emotionally salient information may contribute to the context‐dependent activation of attachment‐related representations and mentalizing biases (see section S2 in supporting information). The LC–NA system may therefore provide a neurobiological link between attachment‐related responses, mentalizing, and emotion regulation by regulating arousal and thereby influencing the conditions under which controlled mentalizing is maintained or gives way to more automatic responding under stress.

Alongside regulation of arousal, the LC plays a central role in the regulation of autonomic function, generally increasing sympathetic activity via activation of ɑ_1_‐adrenoceptors on preganglionic sympathetic neurons and reducing parasympathetic activity via activation of ɑ_2_‐adrenoceptors on preganglionic parasympathetic neurons. Several peripheral and non‐invasive markers of sympathetic and parasympathetic activity have been linked to LC activity and/or emotion dysregulation. Although not specific to LC activity, task‐evoked pupil dilation can provide a reliable proxy of LC–NA system‐mediated neuronal activity, particularly under high arousal states, and is consistently linked to emotional arousal and the recruitment of cognitive resources for goal‐directed activity. Heart rate variability (HRV), which is the variability of the interval between successive heartbeats and considered to be largely dependent on vagal tone and modulation, represents another potential peripheral physiological marker of LC–NA system‐dependent adaptive capacity. Both pupillometry and HRV metrics have been empirically linked to attachment behavior and emotion regulation processes (see sections S3 and S4 in supporting information, respectively), supporting their potential utility as peripheral markers of LC–NA system adaptive capacity and agitation vulnerability.

The LC is the earliest brain region to be affected by AD, as abnormal tau lesions are histologically first detected here in otherwise healthy individuals from early adulthood.^60^ Degeneration of the central NA system is also a pathological hallmark of several other conditions, including Parkinson's disease and Huntington's disease.^61^ There is compelling evidence to support the presence of sustained compensatory LC–NA system functional overactivity as an adaptive response to neurodegenerative processes in AD, particularly in preclinical or early disease stages, which is linked to agitation propensity.^22^, ^23^ LC–NA system‐dependent functional activity likely shows a non‐linear association with LC structural integrity across AD disease stages,^25^ and early AD‐related abnormal tau accumulation in LC neurons, prior to cell loss, may already contribute to changes in emotion processing in preclinical AD individuals. This may be through impaired adaptive tonic and phasic activity patterns that, directly and/or indirectly, push NA‐responsive (PFC and subcortical) systems beyond their optimal dose–response curve^25^ in certain contexts. In other words, early compensatory LC activity may arise from mid‐life and functions to maintain some aspects of cognitive and behavioral performance (e.g., via a rightward shift on the inverted U dose–PFC response curve), but this ultimately reflects aberrant processes that may also have negative consequences for other domains and/or be insufficiently adaptive if contextual demands or stress are too high.^62^

LC–NA system‐dependent processes may therefore intersect with socio‐emotional functioning, including the activation of attachment‐related representations and shifts between mentalizing and non‐mentalizing states under conditions of emotional arousal. In the context of neurodegeneration, altered LC–NA system function in care recipients may impair adaptive emotion regulation, while caregiver mentalizing may be vulnerable to the interpersonal stress and burden associated with progressive cognitive and behavioral change in the care recipient. These complementary processes may interact within the caregiving relationship to influence individual differences in agitation propensity (Figure 1).

## 4 FUTURE RESEARCH DIRECTIONS

The neurorelational model suggests complementary therapeutic targets in care recipients and caregivers, reflecting the different ways in which neurodegeneration and relational stress may affect adaptive emotion regulation. Mentalizing may support adaptive emotion regulation in both groups, with LC–NA function potentially contributing to the arousal regulation required to sustain this capacity. In care recipients, LC–NA function is affected by neurodegenerative processes, providing a potential target for pharmacological modulation, while disease progression increasingly constrains the feasibility of directly modifying mentalizing capacity. In caregivers, by contrast, mentalizing capacity is not directly affected by neurodegenerative processes, making psychosocial approaches to strengthen mentalizing, particularly during agitation‐related interactions, a more feasible intervention strategy.

### 4.1 LC–NA system‐based approaches

For individuals with dementia‐related agitation, LC–NA system dysfunction represents a potential pharmacological therapeutic target, given its role in arousal regulation and adaptive responding. There is some evidence supporting the effectiveness of noradrenergic‐dampening agents such as prazosin,^63^ propranolol,^64^ and dexmedetomidine,^65^ to treat agitation in dementia. However, the overall evidence base is limited, with few small randomized, controlled trials (RCTs) and insufficient consistent high‐quality RCT evidence to support routine clinical use across agents. Evidence that reducing overactivity/responsiveness may improve agitation, while targeting underactivity/responsiveness may improve apathy,^66^ supports the role of baseline LC–NA system capacity in contributing to distinct neuropsychiatric profiles and predicting treatment response and adverse effects in AD. Within this framework, apathy may be understood as reflecting the lower end of LC–NA responsivity, consistent with catecholaminergic contributions to motivation and goal‐directed behavior. Although distinct from attachment‐related deactivation strategies, apathy may share features of reduced environmental engagement, potentially involving overlapping NA mechanisms that warrant further investigation.

### 4.2 Mentalization‐based approaches

The potential benefit of improving mentalizing capacity on neuropsychiatric symptoms in dementia is unknown and, in individuals with more severe dementia, any improvements may be difficult to sustain given the progressive effects of neurodegeneration. Thus, although care‐recipient mentalizing may be relevant to agitation, its progressive decline may limit its potential as a direct therapeutic target. A complementary approach is to optimize caregiver mentalizing, which may remain more modifiable and could help mitigate the effects of neurodegeneration on the care recipient's “neuropsychiatric reserve” by reducing escalation of interpersonal distress. This may, in turn, reduce expressed emotion, agitation, and caregiver distress or psychopathology, and potentially reducing the likelihood of subsequent escalation of care. This approach may be particularly relevant in “at‐risk” caregivers or dyads with lower reflective functioning and other factors that may impair mentalizing (e.g., lower levels of baseline attachment security, comorbid psychiatric disorders, or high perceived levels of stress and arousal). In care recipients, adjunctive physiological or cognitive–affective markers that index LC–NA system functioning may help characterize interindividual differences in LC–NA adaptive capacity and identify those who may benefit from targeted intervention.

While mentalization‐based understandings have been used to support family caregivers in dementia,^67^ and emerging mentalization‐informed interventions (e.g., imagery‐based approaches) have examined related neurobiological and functional outcomes,^68^, ^69^ to our knowledge, mentalizing or reflective functioning has not been directly evaluated as an intervention outcome in dementia caregiver studies. The closest comparable evidence comes from mentalization‐based interventions in parent–child dyads, which have typically focused on enhancing parental reflective functioning through group‐based or dyadic formats that encourage caregivers to reflect on their own and their child's mental states.^70^

For any psychosocial intervention, levels of complexity, intensity, and associated costs need consideration, as these present potential barriers to widespread implementation of existing evidence‐based person‐centered interventions for agitation, particularly those that are integrated with activities and pharmacological components. Clinically significant agitation in populations of people with dementia tends to be driven by a subgroup of individuals,^3^, ^11^ who may be less likely to participate in and more likely to drop out from a program that relies heavily on structured activities, supporting the need to identify and target individuals with higher agitation propensity and support caregiver‐led approaches. Dementia care mapping is an effective person‐centered intervention for agitation in certain contexts, but it is complex and relies on a supportive existing culture and structure of the care home for successful implementation. In long‐term care settings, directly supervised person‐centered training approaches may be more effective than non‐ or indirectly supervised interventions for agitation.^50^ Less intensive psychoeducational approaches can improve community caregiver outcomes such as caregiver burden and depressive symptoms, and reduce relational conflict when delivered to both family caregivers and patients, but their effectiveness for clinically significant agitation symptoms is unclear.

It is possible that any mentalization‐based intervention will need to be adapted for caregivers in different settings, for example, family caregivers at home versus professional caregivers in long‐term care settings, and a range of intensities may be needed to address varying levels of reflective functioning in the caregiver or dyad, for example, psychoeducational versus structured and supervised interventions. To our knowledge, no studies have investigated the effectiveness of targeting caregivers’ understanding of, or capacity for, mentalizing on caregiver psychosocial outcomes (e.g., burden, distress) or care‐recipient agitation propensity. The provision of more intensive interventions to enhance reflective functioning in caregivers or dyads identified as likely to obtain the most benefit, for example, those who exhibit lower reflective functioning scores, could represent a targeted strategy to mitigate agitation propensity and associated caregiver distress.

### 4.3 Knowledge gaps to be addressed

The integrated neurobiological and relational model of agitation propensity, as represented in Figure 1, identifies key gaps in empirical evidence. For example, no studies have investigated whether the observed bidirectional association between caregiver burden and agitation risk could be mediated or mitigated by optimizing caregivers’ mentalizing capacity. While current clinical interventions include both non‐pharmacological and pharmacological approaches, there remains a need to clarify how pre‐morbid emotion regulation capacities can enhance or modify treatment effectiveness. In addition, whether and how mentalization‐based approaches, which have so far been mainly successfully delivered to and evaluated in caregiver–infant and child and psychiatric patient populations, can be effectively adapted for delivery to family or professional caregivers of individuals with dementia needs further study. It would be important to understand how baseline LC–NA system capacity, which may vary within and between disease stages, might influence agitation treatment response with NA‐lowering agents. Future research may clarify whether markers of LC–NA adaptive capacity could contribute to identifying individuals or caregiver–care recipient dyads with relatively lower “neuropsychiatric reserve” and increased vulnerability to agitation. Additionally, given the complex and multifaceted role of the LC–NA system and the involvement of other neurotransmitters, research should explore broader neurochemical influences on agitation and their interaction with neuropsychiatric reserve. Concurrently, studies should aim to validate LC–NA system‐dependent functional measures, such as pupillary measures and HRV indices, as putative markers of agitation propensity.

Such physiological markers, however, are influenced by age, comorbid medical conditions, and medications common in older adults, which future research will need to carefully control for or incorporate into study designs. To our knowledge, no studies have evaluated the comparative efficacy and safety of different NA‐lowering agents in reducing agitation propensity, and although the impact of pharmacological interventions on caregiver psychosocial outcomes is considered a meaningful outcome,^71^ these are not typically included as primary endpoints and are not central to regulatory evaluation.

Experimental medicine studies could investigate the impact of NA (vs., e.g., dopaminergic) agents on relevant processes, such as the modulation of attention and response inhibition in the context of increasing levels of emotional arousal, in older individuals who have different levels of reflective functioning, AD pathology, and agitation propensity. It would also be important to investigate the relationship between reflective functioning, emotion regulation adaptivity, and psychosocial outcomes in caregivers in prospective cohort studies. These studies could test the hypothesis that central NA system overactivity contributes to agitation through impaired emotion regulation and heightened threat reactivity, and/or that lower caregiver mentalizing capacity predicts increased agitation in people with dementia over time. Addressing these knowledge gaps could support the development of personalized neurorelational approaches to treat agitation, comprising mentalization‐based interventions of varying intensity levels for caregivers and/or NA‐lowering interventions for care recipients, that can be evaluated in community and long‐term care settings.

## 5 CONCLUSION

Distressing neuropsychiatric symptoms such as dementia‐related agitation, which arises in the context of interpersonal interactions, requires an integrated therapeutic approach that includes neurobiological and relational perspectives and considers the experiences of both care recipients and caregivers. This review hypothesizes that care recipients’ LC–NA system functional capacity and caregivers’ reflective functioning could serve as potential mechanisms underlying agitation, which may inform future research and the development of intervention strategies. Future studies are needed to refine and validate the neurorelational model of agitation propensity and evaluate interventions that could mitigate the effects of neurodegeneration on “neuropsychiatric reserve.”

## CONFLICT OF INTEREST STATEMENT

The authors declare no conflicts of interest. Author disclosures are available in the Supporting Information.

## Supporting information



Supporting Information

Supporting Information
