# Supplementary material for: Advancing therapeutic approaches for agitation: A neurorelational perspective
Source: Alzheimers Dement (N Y). 2026 Oct 14;12(4):e70333. doi: 10.1002/trc2.70333 (PMC13662826; doi:10.1002/trc2.70333)
Supplement: Supplementary file 2 [file TRC2-12-e70333-s002.docx]

**Supplementary Materials**

[Section 1	2](#_Toc219373179)

[Section 2	2](#_Toc219373180)

[Section 3	3](#_Toc219373181)

[Section 4	3](#_Toc219373182)

[REFERENCES	4](#_Toc219373183)

# Section 1

Agitation in both cognitively normal Alzheimer’s disease (AD)-biomarker positive and cognitively impaired individuals with AD is related to tau-mediated pathology detected using PET [1], CSF [2] and plasma [3], while increasing AD-related pathological tau load increases the likelihood of agitation relative to other neuropathologies [4]. As well as possible direct effects of AD neuropathology on emotion regulation processes, reduced metabolic activity [5], concomitant cerebrovascular disease (CVD)[6] or microglial activation [7] in emotion regulation regions may also contribute to agitation severity in mild cognitive impairment (MCI)/AD dementia.

# Section 2

The level and mode of locus coeruleus (LC) activity is influenced by a balance of neuromodulatory influences, including endogenous opioids and corticotropin-releasing factor (CRF), which are involved in attachment behavior [8] and have opposing effects on LC activity. Endogenous opioids have an inhibitory influence and shift LC activity away from a high tonic and towards a phasic mode, whereas CRF has an opposite excitatory effect and LC neurons can become sensitized to low levels of CRF in response to stress [9]. The LC-noradrenaline (NA) system’s key role in emotional memory, involving the selective encoding of higher versus lower priority representations, is amplified via local glutamate activity ‘hotspots’ [10], that can potentially be selectively reactivated upon encountering a contextually similar situation. This could potentially underlie contextually activated attachment representations or trigger the retrieval of (biased) assumptions associated with automatic mentalizing states.

# Section 3

LC-NA responsivity, as indexed by pupillometry [11], predicts resilience to real-world stress and psychopathology [12] and has been linked to attachment behavior systems, i.e. attachment anxiety showed greater pupil dilation during cognitive reappraisal, consistent with heightened cognitive effort and hypervigilance, whereas attachment avoidance showed the opposite pattern with reduced pupil dilation [13].

# Section 4

Consistent with the expectation that higher LC activity would be associated with lower heart rate variability (HRV) through sympathoexcitatory and parasympatholytic effects, in vivo imaging studies in healthy adults have shown an inverse relationship between resting high-frequency HRV (considered to most represent ‘vagally-mediated’ HRV) and LC MRI contrast ratios (a measure of LC structural integrity) [14] and LC fMRI BOLD activity [15]. More broadly, HRV is proposed to index the functional capacity of prefrontal regions supporting self-regulatory and socio-emotional processes that underlie emotion regulation [16], and higher HRV is associated with the activation of secure versus insecure attachment representations [17] in response to stress. Functional neuroimaging correlates of both HRV and emotion regulation have consistently included fMRI activation of medial PFC, amygdala and other regions proposed to form a central autonomic network [18]. Consistent with a bidirectional connection between cardiac and LC activity via the vagus nerve, HRV biofeedback interventions may modulate emotional processing in PFC regions [19]. Although most studies have measured HRV at rest (‘tonic’ HRV) compared to the response to an event or task (‘phasic’ HRV), measures of HRV reactivity during, and in recovery after stressful or salient stimuli relative to baseline, i.e. autonomic system flexibility [20], may provide further insights on individual differences in adaptive capacity.

# REFERENCES

[1]	Naude J, Wang M, Leon R, Smith E, Ismail Z. Tau-PET in early cortical Alzheimer brain regions in relation to mild behavioral impairment in older adults with either normal cognition or mild cognitive impairment. Neurobiol Aging 2024;138:19–27.

[2]	Bloniecki V, Aarsland D, Cummings J, Blennow K, Freund-Levi Y. Agitation in dementia: relation to core cerebrospinal fluid biomarker levels. Dement Geriatr Cogn Dis Extra 2014;4:335–43.

[3]	Krell-Roesch J, Zaniletti I, Syrjanen JA, Kremers WK, Algeciras-Schimnich A, Dage JL, et al. Plasma-derived biomarkers of Alzheimer’s disease and neuropsychiatric symptoms: A community-based study. Alzheimers Dement 2023;15:e12461.

[4]	Devanand DP, Lee S, Huey ED, Goldberg TE. Associations Between Neuropsychiatric Symptoms and Neuropathological Diagnoses of Alzheimer Disease and Related Dementias. JAMA Psychiatry 2022. https://doi.org/10.1001/jamapsychiatry.2021.4363.

[5]	Ng KP, Pascoal TA, Mathotaarachchi S, Chan YH, Jiang L, Therriault J, et al. Neuropsychiatric symptoms are early indicators of an upcoming metabolic decline in Alzheimer’s disease. Transl Neurodegener 2021;10:1.

[6]	Anor CJ, Dadar M, Collins DL, Tartaglia MC. The Longitudinal Assessment of Neuropsychiatric Symptoms in Mild Cognitive Impairment and Alzheimer’s Disease and Their Association With White Matter Hyperintensities in the National Alzheimer’s Coordinating Center’s Uniform Data Set. Biol Psychiatry Cogn Neurosci Neuroimaging 2021;6:70–8.

[7]	Schaffer Aguzzoli C, Ferreira PCL, Povala G, Ferrari-Souza JP, Bellaver B, Soares Katz C, et al. Neuropsychiatric Symptoms and Microglial Activation in Patients with Alzheimer Disease. JAMA Netw Open 2023;6:e2345175.

[8]	Izaki A, Verbeke WJMI, Vrticka P, Ein-Dor T. A narrative on the neurobiological roots of attachment-system functioning. Commun Psychol 2024;2:96.

[9]	Valentino RJ, Van Bockstaele E. Convergent regulation of locus coeruleus activity as an adaptive response to stress. Eur J Pharmacol 2008;583:194–203.

[10]	Mather M, Clewett D, Sakaki M, Harley CW. Norepinephrine ignites local hotspots of neuronal excitation: How arousal amplifies selectivity in perception and memory. Behav Brain Sci 2016;39:e200.

[11]	Joshi S, Li Y, Kalwani RM, Gold JI. Relationships between Pupil Diameter and Neuronal Activity in the Locus Coeruleus, Colliculi, and Cingulate Cortex. Neuron 2016;89:221–34.

[12]	Grueschow M, Stenz N, Thörn H, Ehlert U, Breckwoldt J, Brodmann Maeder M, et al. Real-world stress resilience is associated with the responsivity of the locus coeruleus. Nat Commun 2021;12:2275.

[13]	Domic-Siede M, Guzmán-González M, Sánchez-Corzo A, Granillo L, Salazar C, Silva M, et al. Pupil responses to emotion regulation strategies: the role of attachment orientations. Cogn Emot 2025:1–18.

[14]	Bachman SL, Nashiro K, Yoo H, Wang D, Thayer JF, Mather M. Associations between locus coeruleus MRI contrast and physiological responses to acute stress in younger and older adults. Brain Res 2022;1796:148070.

[15]	Jacobs HI, Priovoulos N, Poser BA, Pagen LH, Ivanov D, Verhey FR, et al. Dynamic behavior of the locus coeruleus during arousal-related memory processing in a multi-modal 7T fMRI paradigm. Elife 2020;9. https://doi.org/10.7554/eLife.52059.

[16]	Smith R, Thayer JF, Khalsa SS, Lane RD. The hierarchical basis of neurovisceral integration. Neurosci Biobehav Rev 2017;75:274–96.

[17]	Bryant RA, Hutanamon T. Activating attachments enhances heart rate variability. PLoS One 2018;13:e0151747.

[18]	Mulcahy JS, Larsson DEO, Garfinkel SN, Critchley HD. Heart rate variability as a biomarker in health and affective disorders: A perspective on neuroimaging studies. Neuroimage 2019;202:116072.

[19]	Min J, Rouanet J, Martini AC, Nashiro K, Yoo HJ, Porat S, et al. Modulating heart rate oscillation affects plasma amyloid beta and tau levels in younger and older adults. Sci Rep 2023;13:3967.

[20]	Lin FV, Heffner KL. Autonomic nervous system flexibility for understanding brain aging. Ageing Res Rev 2023;90:102016.
